# Factors Influencing Consumers’ Purchase Intention Towards Green Home Appliances

**DOI:** 10.3389/fpsyg.2022.927327

**Published:** 2022-06-30

**Authors:** Chen Wei Teoh, Kok Chin Khor, Walton Wider

**Affiliations:** ^1^School of Business, INTI International College Penang, Penang, Malaysia; ^2^Faculty of Business and Communication, INTI International University, Nilai, Malaysia

**Keywords:** consumer purchase intention, after sales service, brand equity, environmental awareness, green home appliance

## Abstract

The main purpose of this study is to investigate the effect of after sales service (ASS), brand equity (BE), environmental awareness (EA), and product pricing (PP) towards consumer purchase intention (CPI) of green home appliance. Data were collected from 150 Penang, Malaysia citizen who were age from 18 to 60 *via* convenient sampling method analysed using partial least square structure equation modelling (SmartPLS). Results indicated that BE, EA, and PP has significant effect on CPI of green home appliance brand. However, ASS do not have significant effect on CPI on green home appliance brand. This research helps home appliance manufacturer to better design marketing promotions, considering the consumers focus on BE, EA, and pricing. In addition, this study provides consumer insight for the government agency to construct better policy in order to increase the green home appliance penetration rate among citizens.

## Introduction

The term green home appliance generally is used to describe any energy-efficient home appliance, they are designed to increase energy efficiency of the electric device and reduce utility bills. There is a wide array of green household appliances such as air-conditioning, water heater, lighting, washing machine, refrigerators, oven, television, and many more. The more efficient the appliance, the less energy it will use. Air conditioning, for example, with inverter and remote sensor technologies, it reduces electricity by 25–30% (King and Perry, 2017). There are different factors to take into consideration, including efficiency, price, and energy labelling by local authority. Green product has become a market mainstream contemporarily in developed and developing countries. The level of awareness in the society highly depends on initiative by the government and non-government bodies *via* education system and social campaign. World energy consumption is growing exponentially, and energy conservation become a huge initiative by government and business stake holders to keep the environment sustain (Singhal et al., 2019). All energy consumption comes with an environmental cost, especially fossil fuel generation (Al-Marri et al., 2018). Among all sector, residential energy consumption has an important role to play in the entire energy consumption outcomes and it is intricately linked with consumer behavioural, especially the buying intention of green home appliance. Consumer intention is hard to predict as it involves comprehensive psychological processes. During a purchasing process, a consumer will initiate with recognizing the needs, finding way to resolve, interpret information, evaluating multiple choice, and finally making the decision to purchase a specify product (Singh and Verma, 2017). Various studies attempt to understand the determinants or stimuli associate to the purchase intention behaviour. Subsequently, help the company to frame out effective marketing strategies and plans.

From a marketing perspective, by understand the consumer intention underlying the decision to purchase green home appliance, it helps to facilitate the promotion and sales of the product. As the price of green home appliance is always higher relatively compare to traditional home appliance, individual which buy the green home appliance need to have the perceived values rather than the product primary functions. This derive that the contemporary green values of the products are more than their fundamental functions (Jan et al., 2019). Generally, environmental impacts are an inherent part of electricity production and energy use. Therefore, green home appliance plays an essential role in helping to reduce the use of electricity, and subsequently contribute towards the environment sustainability. Zhang et al. (2020) revealed that household appliances such as air conditioners, washing machines, and refrigerators are responsible for high household energy consumption and carbon emission in China. The use of energy-efficient appliances has an important positive impact on reducing household energy consumption and household carbon emissions (Zhang et al., 2020).

In the context of Malaysia, the utilization of green home appliance among Malaysia consumer is not promising (Tan et al., 2019). Their study reveals that only 30% of the respondents has the experience of purchasing green product. This deduces that other determinants have influenced the consumers for not purchasing green home product, for example, product pricing (PP), product quality, and product branding. In most Malaysians perspective, they assumed that green products as highly priced goods, even with additional attributes comparison with non-green products (Khor and Mah, 2020). According to Malaysia Energy Commission (2017), in the period of 1990–2016, more than 90% of electricity generated for Peninsular Malaysia was fossil fuel. In 2016 alone, for example, coal provided up to 52% of the energy generation while gas contributed 44%, these methods have significant impact on the environment.

Study show that by increasing the willingness to purchase energy-efficient appliances is vital to reduce household energy consumption and carbon emissions resulting from this daily consumption (Li et al., 2019). Energy-efficient home appliances produced an annual electricity savings of about 10 billion kWh, which was equivalent to a reduction of carbon emissions by 6.5 million tons (Li et al., 2021). Back to the context of Malaysia, the penetration of green home appliance among Malaysia consumer are less than 30% (Tan et al., 2019), while the electricity generation in Peninsular Malaysia are origin from fossil. Thus, it denotes the huge potential in Malaysia for the energy and environment conservation. This can be achieved if we manage to enhance consumer environment awareness to utilize energy saving appliance in their daily lifestyle. In Iran, the local home appliance brands failed to compete with the foreign brands, even Iran manufacturers has the similar production technology and capacity (Esmaeilpour, 2014). The citizens in Iran prefer to buy home appliance origin from foreign country like Japan and Europe. The research reveals that undesirable after sales service (ASS) has reduced the interest Iran citizen to purchase a local product and decided to shift to other foreign brand. In the context of Malaysia, similar scenario in automobile industry. Proton, once the leading car seller in Malaysia market has a cliff fall since early 2000, one of the reasons is due to the poor ASS which hinder existing customer to continue support the automobile company (Saidin et al., 2015). Poor ASS by Proton, followed by criticism in social media by existing customer has result in the tendency of potential customer swing to other choice. Two scenarios above show that poor ASS is an impediment to the purchase of product. Meanwhile, the price for green home appliance is relatively high compare to normal home appliance. This could be another hurdles and barrels to increase the penetration of green home appliance product among consumers as the purchase power has significant correlation with income per capital. If the research able to overcome the price gap, there was a huge potential market among low (B40) and medium-income (M40) customers, who represent 80% of Malaysia population (Ng et al., 2018).

The variables focused in this study are the core marketing processes such as ASSs, brand equity (BE), EA, and PP where research previously published is not extensive in Malaysia (Surianshah, 2021). Therefore, this study aims to answer the research question, “What are the factors influencing on consumer purchase intention (CPI) of green home appliance?” Answering this question will help home appliance manufacturer to better design marketing promotions. In addition, it is hoped that the findings from this study will provide consumer insight for the government agency to construct better policy in order to increase the green home appliance penetration rate among citizens.

## Theoretical Underpinnings

The foundation for the study is theory of planned behaviour (TPB). The attitude, subjective norm, and perceived behavioural control are three core components in TPB, which together devise an individual’s behavioural intentions (Ajzen, 2015). Many studies generally assume the intentions are good predictors of behaviour and fully mediate the impact of attitude and subjective norm towards the behaviour (Ashraf, 2021). In addition, Hutahaean and Kurnia (2020) declared that intention is still accepted as the best available predictor of behaviour. Additionally, attitude, subjective norms, and behaviour control both have significantly positive impacts on consumer intention (Lin and Guan, 2021). It helps reader to understand how the behaviour of consumer can change by their beliefs how a certain green product could satisfy their needs. Superficially, the TPB is widely applied behavioural model in green marketing research (Stavros et al., 1999; Prigita and Alversia, 2022). However, as asserted by Harun et al. (2022), the literature on green purchasing behaviour is still in infancy in the context of South East Asian countries especially in Malaysia; furthermore, researchers have just begun to adopt TPB in green purchase intention of green home appliance in recent years. Therefore, the current study aims to fill in the research gap by directly enriching the literatures.

Studies denote that TPB reconciling the relationship between environment concern and green product buying intention (Afridi et al., 2021). They also concluded that TPB demonstrate higher utility in predicting consumer green purchase intention. These include green hotel and restaurant (Han, 2020); and green organic food (Pacho, 2020). In addition, findings indicated that the subjective norm was found to have the highest direct influence on purchase intention of green apparel in United States and China. Individuals may feel more pressure from their relevant groups when they conduct environmental behaviours, such as green consumption, recycling, and eco activities that may require ethical and socially responsible responses (Ko and Jin, 2017). Hence, the fundamental theory and conceptual framework for this research were devised based on the TPB.

## Literature Review

### The Influence of After Sales Service on Consumers’ Purchase Intention

After sales service can be define as activities occur after the purchased of a particular product, and the devotion of the seller to the customer to assure the functionality of the product (Murali et al., 2016). Besides, ASS may be defined as the various processes with the main objective is to make sure customers are satisfied with the products and services of the organization (Domazet et al., 2018). In other words, ASS are important part of total customer satisfaction. Service quality is a measure of how well the service level delivered matches customer expectations and also a vital indicator for satisfaction (Yadav, 2019). Customers of green home appliances are more demanding nowadays and expecting impeccable ASS support. Especially, when they need some emergency services after the sale, the service would be fast and the costs are fairly accessible (Murali et al., 2016). Due to the fact that literatures on the influence of ASS on green purchase intention is scarce, it is difficult for the researcher to discussed based on green marketing perspective. Nevertheless, previous studies have revealed that consumers always consider the functionality and durability of the home appliance in their purchase intention, durability is evidenced has correlation with ASS (Esmaeilpour, 2014). Past research identified warranty accessibility and support as a key dimension of ASS as warranty provides assurance of quality of the purchased green home appliance, where longer warranty period assures a higher quality and reliability the product (Wickramasinghe and Mathusinghe, 2016). Irrefutably, household appliances are relatively expensive products and plenty consumers are obliged financial consequences during the purchase of home appliance like refrigerator, washing machine, and air conditioning.

According to Seth et al. (2005), ASS are recognised as a primary determinant which impact the CPI. In long term, it contributes to the revenue, profit, and competitive advantage for many industries. However, most of the business organizations are not aware about the ASS factors. Failing to understand the pivotal of ASS can lead to a disastrous to the business organization. The negative word-of-mouth effect will cause a potential customer switch to other competitor during the selection of green home appliance and vice versa (Wickramasinghe and Mathusinghe, 2016). Company should know the objective and importance of having ASS and implement it to satisfy customers, retain them and enhance their loyalty in next purchase. Becoming similar in term of attributes for green home appliance product, the ASS reputation can effect on consumers’ way of choice (Abdullah and Hilmi, 2014). It represents a significant source of competitive advantage for the companies that operate in the same market. Similar thoughts presented by Posselt and Gerstner (2005) that the repurchase intention has significantly influence by the ASS, especially consideration on the spare part availability, optimization of warranty period and scope of work during warranty period.

After sales service attributes have significant impact in building customer trust and satisfaction, subsequently result in customer upcoming purchase intention (Murali et al., 2016). On the meta-analysis study of the purchase intention of the consumers towards remanufactured products conducted by Singhal et al. (2019), they revealed that ASS is moderately affecting the consumer buying intention. A structural empirical analysis done in Beijing on the factors influencing Electrical Vehicle (EV) purchasing intention. The findings reported that ASS is one of the consideration of CPI during the selection of EV (Huang and Ge, 2019). An investigation on the importance of ASS on three leading home appliances manufacturing firms in South India revealed that the ASS practices has a positive effect on the household appliances industry (Murali et al., 2016). While in Serbia, the warranty coverage period and the ASS accessibility are recognised as critical criterion which will define the customer purchase intention on particular appliance product especially washing machine, refrigerator, and air conditioning (Domazet et al., 2018). Furthermore, Vanniarajan (2011) revealed that the ASS quality attributes have significant influence on the repurchase intention of customer in electronic good market in India. Hence, this research would like to examine if the ASS is substantial factor influencing CPI towards green home appliance in Malaysia. specifically, we hypothesised that:

*H1: After sales service positively affects the purchase intention of green home appliance brand.*

### The Influence of Brand Equity on Consumers’ Purchase Intention

Brand equity is exhibit an added value to a brand and product and such a value is made of customer’s positive feelings, thinking, and acting towards purchasing a brand (Senthilnathan and Tharmi, 2012). Branding is a very practical strategy for differentiating product or service in the specify industries. It is a special value given to a product through its name. Successful brands allow company to gain competitive advantage including the opportunity for successful extensions, resilience against competitors’ pricing strategies, and the ability to create barriers to rivalry entry (Seitz et al., 2010). In addition, a powerful brand influences attitudes of customers and makes a strong product association through the brand. Consumer will demonstrate different reaction toward a main brand and a product without a brand even both have the same features and functionality (Khan et al., 2015). According to Aaker (1996), the BE is a multi-dimensional concept which include brand awareness, perceived quality, brand association, and brand loyalty. Understanding consumers’ purchasing behaviour enable the company to attract and retain customers. Research reveal that strong brand awareness and association has significant impact on CPI (Gautam and Shrestha, 2018). By paying more attention to BE, it helps to increase the purchase intention of consumer.

Research indicates that the higher the BE the stronger consumers’ preferences and purchase intentions (Moreira et al., 2017) to obtain the product or service. Aaker (1996) has proven that the benefit of BE in creating positive attitude among consumers, generating their interest to buy the product by distinguishing the brand within the same sector. This statement is further strengthened by the research in automobile industry where the brand perceived quality, brand loyalty and brand awareness have strong correlation with consumer buying intention (Jalilvand et al., 2011). A study in the United States argued that BE is not a main consideration during CPI on air conditioning, as quality of product is their primary choice of criterion (Seitz et al., 2010). The study in smartphone industry found that BE has positive association with the purchase intention as the signage of specify brand, for example, Apple provide user the status in society (Akkucuk and Esmaeili, 2016). Whereas in food industries, investment in BE enable marketer to elevate the consumer buying intention (Roozy et al., 2014).

Besides, the findings in Malaysia fashion industry reveal that the is casual relationship among four dimensions of BE to CPI. Brand loyalty has stronger impact on purchase intention among the four segment in this study (Khan et al., 2015). Moreover, on the baby care product segment in Sri Lankan context where parents’ safety consciousness is augmented. It demonstrated positively significant linear relationship between BE and purchase intention (Senthilnathan and Tharmi, 2012). Studies in smart phone industry in developed country evidenced a strong positive correlation between BE with purchase intention. This can be explained that consumer has familiar with the product brand, and quality assurance, and social status (Huang et al., 2019). A research carried in Iran exhibit that BE not only has positive association with purchase intention, it has the globalization impact where brand which is highly rated in their country will received the same acceptance in other country (Moradi and Zarei, 2011). Further study on Samsung home appliance in Ishafan, Iran deduce that user experience has the greatest impact among all the effective dimension under BE which motivate CPI to home appliance. This past experience dimension is built over the time (Shafiee et al., 2019). Moreover, Vazifehdust et al. (2017) reported that there are significant relationships between BE and ultimate consumer buying intention on home appliance. Therefore, the current study would like to examine if the BE is pivotal factor influencing CPI towards green home appliance in Malaysia. specifically, we hypothesised that:

*H2: Brand equity positively affects the purchase intention of green home appliance brand.*

### The Influence of Environmental Awareness on Consumers’ Purchase Intention

Environmental awareness is defined as environmental awareness refers to the concerns and comprehension of environmental problems (Chen et al., 2019). The attitude of consumer will be influenced positively by EA and significantly impact on the purchase intention on green product (Akroush et al., 2019). Consumer who has green consciousness and believe that their approach will help the environment benefit, will demonstrate a green product purchase intention. However, it they believe their approach cannot lead to a positive change or it is not worth the price, they will remain inactive (Maniatis, 2016). In additional, research exhibit that the energy concern will drive consumers’ ethical commitments and further motivate them to green product (Ha and Janda, 2012).

According to Franzen and Meyer (2010), consumer who has high awareness of environmentally and energy concern, will be more alert on the natural resource’s utilization on our planet. Green awareness influences human behaviour in several ways, which include reducing consumption, changing wasteful or harmful consumption patterns, and raising preference for environmentally friendly products (Suki, 2013). The social responsibility towards environment able to motivate the buying behaviour among consumers (Tan et al., 2019). Consumers who have EA means consumer embrace lifestyle and living activities which have a positive impact on environment or alternatively reduce negative impact on the environment (Wu and Chen, 2014). Research denotes that the green initiatives done by government has significant influence on household consumers in China, the campaigns has driven the green appliance sales for over the precedent year (Li et al., 2019). Nonetheless, the positive purchase attitude by Chinese consumers is believed to be stimulated by the high environment and energy conservation consciousness (Ha and Janda, 2012).

Furthermore, environmental concern and environmental knowledge have positive correlation on the willingness to purchase green products among young consumers when predicting the willingness of young consumers to purchase green products in developing countries (Yadav and Pathak, 2016). Nonetheless, the home appliance manufacturer cannot promote a green product by only focus on environmental benefits and not benefits of the consumers in term of functionality. Consumers who embrace environment friendly product do not differentiate between environmental and economic benefits. They see them as complimentary during the process of fulfil their personal needs as well as environment commitment (Maniatis, 2016).

In addition, the public also need to be assured that any conscious efforts made towards protecting the environment through energy conservation are going to make a difference strategically in the long term. Research show that environmental concern and environmental knowledge have significant association consumer attitude and impact on the purchase intention in TPB model (Li et al., 2019). By increasing the green home appliance penetration rate, it will have a positive correlation with the saving of electricity. Further study has empirically verified the essence of knowledge on environment and impact to the surrounding in the purchase of energy-efficient home appliances (Waris and Hameed, 2020). Research in Qatar further support hypothesis which depicts that consumers have a positive inclination for the purchase of energy-efficient products if consumer is exposed to the information of environment and energy conservation *via* marketing materials (Al-Marri et al., 2018). Furthermore, Ko and Jin (2017) had empirically tested that environmental knowledge has positive correlation with the green apparel consumption. Both United States ad China sampling demonstrated the similar result of environment awareness association. To augmenting consumer awareness of residential energy consumption, United States government has launched the Energy Star Program in 1992. Even, awareness also does not directly translate into the purchase decision of energy saving home appliance, but implicitly assumes that the increased awareness will significantly impact on the consumer intention to shift from non-energy saving appliances to energy saving appliances (Murray and Mills, 2011).

In the context of Malaysia, study by Noor et al. (2012) showed that most of Malaysia acquired moderate environmental issue and knowledge. The study also reported that the environmental concern and attitudes positively contributed to green purchase intention. Teng et al. (2021) also reported that consumers who possessed high environmental concerns more likely to buy green products than consumers’ who do not have concern on the environment. In addition, Ahmed et al. (2019) reported that environmental consciousness is directly influenced the consumer decision to re-purchase green home appliances. Hence, several studies have confirmed that a consumer who has concern for the environment will tend to have a stronger intention in purchasing a green product. In short, this research would like to examine if the awareness of environment and energy consciousness is substantial factor influencing CPI towards green home appliance in Malaysia. specifically, we hypothesised that:

*H3: Environmental awareness positively affects the purchase intention of green home appliance brand.*

### The Influence of Product Pricing on Consumers’ Purchase Intention

Product pricing is one of the important factors in determining consumers’ willingness to pay a premium green product home appliance. There is salient evidence that consumer intention to purchase can be significantly influenced by the presentation of a price (Weisstein et al., 2014). Although the perceived price is studied extensively in different industries, its influence has rarely been studied in the context of energy efficient home appliance industry (Akroush et al., 2019). If companies fail to distinguish themselves from product features, pricing strategies is the most effective way to gain the competitive advantage in the marketplace, and subsequently increasing the revenue (Ansar, 2013). Drozdenko et al. (2011)’s study shows that although that there is a positive relationship between prices and consumer buying behaviour, and consumer willing to pay more if the product perceived value is higher. Nonetheless, several studies reveal that consumer will feel unfair if they could not perceive the difference or additional value in the product they purchased with higher price. Hence, it will lead to the spreading of negative information *via* word of mouth and have adverse effect on the seller (Victor et al., 2018). However, in the context of price fairness, consumer will have positive intention if the potential benefit of purchase green product able to overcome the price gap (Le and Liaw, 2017). Understanding how consumers make up their perceptions of prices is an important marketing strategy. However, there is research argued that most Malaysians perceived green products as highly priced goods, which is a resistance for them to purchase green home appliance (Sharaf et al., 2015). In the context of green home appliance, the purchasing and using energy-saving appliances are economical in the long run. The higher the perceived long-term cost reduction, the higher the perceived price value. The perception of consumer that the use of energy-saving appliances can save money when price value is high need to be incorporated in the marketing strategy (Zhang et al., 2020). The benefit to environment only be a secondary consideration of consumer in their purchase intention (Li et al., 2019).

However, low pricing policy in retail sector signalling positive impact on CPI. The low pricing strategy enable to marketer to build the belief and intention of the consumer on buying a product in discount period. In short, when price dispersion is high, consumers’ purchase intentions are higher (Özer and Zheng, 2016). An appropriate pricing strategy is required during a new product launched, this applies to the green home appliance. Pricing strategy are major tools that company need to devise to persuade consumers to purchase the product in market (Lee, 2014). The pricing of green home appliance is relatively higher compare to traditional home appliance due to the additional attribute and benefits. Studies exhibit that consumer willing to pay extra cost compare to normal green product if there is a return of investment in utility rate (Drozdenko et al., 2011; Liargovas et al., 2017). However, if the level of green awareness is low among the consumers, their willingness to pay more for green products has declined even though they care about environmental issue (Ginsberg and Bloom, 2004; Chaimankong and Chaimankong, 2019).

Further study in hybrid car segment empirically show that the potential fuel saving will positively drive CPI even the pricing is relatively higher to normal vehicle (Chen et al., 2010). However, in electronic gadget sector for instance smart phone (Huang et al., 2019), price sensitivity is found to be one of the critical factors which positively influence CPI. A positive relationship exists between the price and green purchase intention that favours the notion that environmentally conscious individual might buy the environment friendly product even at a higher price (Drozdenko et al., 2011; Ansar, 2013). Moreover, Ko and Jin (2017) in their study which was conducted in both US during winter and China during summer using theory of TPB, summarised that feasible pricing strategy has positive association with green fashion product. Extended from previous studies, this research would like to further examine if pricing has significant influence on CPI on green home appliance. Specifically, we hypothesised that:

*H4: Product pricing positively affects the purchase intention of green home appliance brand.*

## Materials and Methods

### Population

The population targeted in this study is the Malaysian population who are staying in Penang, Malaysia, age between 21 and 60 years old. They are chosen due to this population are the working adult who has the purchase power.

### Sample

There is no rule of thumb that applies to all situations in fact as the sample size needed for a study depends on many factors including the size of the model, distribution of the variables, amount of missing data, reliability of the variables, and strength of the relationships among the variables (Muthén and Muthén, 2002). Nonetheless, there are few complex formulas to decide the sampling size, the general rule of thumb is no less than 50 participants (VanVoorhis and Morgan, 2007).

### Sampling Design

We conducted an online survey was used to recruit participants using a convenience sampling method. In this research, convenience sampling was chosen due to the limitation of Movement Control Order 2.0 in Malaysia.

### Data Collection Procedure

Data collection was held over a 2-week period in January 2021. It should be pointed out that a Movement Control Order (MCO) was being imposed in Malaysia at that time, from January 13, 2021 (Rodzi, 2021). The Malaysian government temporarily stopped or restricted many activities and businesses were closed. Specifically, the employer would have to also decide the total workforce present at the premises so that safe physical distancing can be maintained at the workplace. Due to this restriction, research activities were affected and numerous procedural adjustments had to be made (Weissman et al., 2020). Therefore, to ensure compliance with safety requirements, switching to online data collection was deemed necessary.

Using a convenience sampling technique, the participant fulfilling the inclusion criterion was selected to complete the self-report survey administered *via* the Internet, through Email and WhatsApp. A total of 150 responses were received from respondents, of which all responses were usable. The response rate for the study was 100%. A total of 150 responses were collected for this study. In addition, to determine the minimum required sample size in terms of statistical power, we utilised G*Power (Faul et al., 2009). The model of this study had four predictors. By using G*Power with an effect size of 0.15, alpha of 0.05, and a power of 0.95, the minimum sample size needed was only 129. Thus, we can safely say that our study with a sample size of 150 has a power of more than 0.95 and is large enough, and the findings can be used with confidence.

Table 1 demonstrated demographic of the respondents where 69.5% are male and 30.5% are female. Majority of the respondents are come from age group of 31–40 with 41.7%, followed by 26–30 with 27.8%; 41–50 with 19.2%; 51–60 with 9.9%; and 18–25 with 1.3%, for educational level, majority hold bachelor degree with 78.8%, followed by High School Level with 13.2%, others with 5.3%, and Master’s degree with 2.6%, for marital status, most of the participants are married with 60.3%, followed by single with 38.4%, divorced and widowed are 0.7%, respectively.

**TABLE 1:** Demographic profile of respondents.

	Frequency	%
**Gender**		
Male	105	69.5
Female	46	30.5
**Age**		
18–25 years old	2	1.3
26–30 years old	42	27.8
31–40 years old	63	41.7
41–50 years old	29	19.2
51–60 years old	15	9.9
**Ethnicity**		
Malay	113	74.8
Chinese	21	13.9
Indian	17	11.3
**Education level**		
High school	20	13.2
Bachelor degree	119	78.8
Master’s degree	4	2.6
Others	8	5.3
**Marital status**		
Single	58	38.4
Married	91	60.3
Divorced	1	0.7
Widowed	1	0.7

## Measures

### Purchase Intention

This construct comprises 5 items which are adapted from Lee (2014) using the 5-Point Likert Scale, ranging from 1 (*strongly disagree*) to 5 (*strongly agree*). The following items were used: “I am going to purchase green home appliance”; “I am planning to purchase green home appliance”; “I am likely to purchase green home appliance”; “I will purchase green home appliance in future”; and “I will purchase again green home appliance in future.” The Cronbach’s alpha (CA) coefficient was 0.91.

### Environmental Awareness

This construct comprises 4 items adapted from Suki (2013) using the 5-Point Likert Scale, ranging from 1 (*strongly disagree*) to 5 (*strongly agree*). The following items were used: “I agree that environmental issue is an emergency issue”; “I agree that environmental issues are consumers’ responsibility”; “My daily activities affect the environment”; and “I agree that buying a green product, indirectly influence the environmental.” The CA coefficient was 0.82.

### After Sales Service

This construct comprises 5 items adapted from Muraly (2019) using the 5-Point Likert Scale, ranging from 1 (*strongly disagree*) to 5 (*strongly agree*). The following items were used: “I expect the company ASS staff is dependable and consistent in solving customer complaints”; “I expect the company ASS staff is fast to respond”; “If any delay in repair service/replacement on my product purchased always update me within short period”; “I expect easy accessible towards the service centre”; and “I expect the company ASS staff is polite and knowledgeable.” The CA coefficient was 0.93.

### Brand Equity

This construct comprises 5 items adapted from Jalilvand et al. (2011) using the 5-Point Likert Scale, ranging from 1 (*strongly disagree*) to 5 (*strongly agree*). The following items were used: “I can quickly recall the logo or symbol of a specify Home Appliance brand”; “Some characteristics of Home Appliance Brand X come to my mind quickly”; “I am aware of Brand X”; “I will not buy other brands, if X brand is available”; and “I always perceive Brand X must be of very good quality.” The CA coefficient was 0.91.

### Product Pricing

This construct comprises 5 items adapted from several sources using the 5-Point Likert Scale, ranging from 1 (*strongly disagree*) to 5 (*strongly agree*). The following items were used: “I am willing to pay extra for recycle product,” “I am willing to pay extra for energy saving product,” and “I am willing to pay extra with if government tax benefit” were adapted from Drozdenko et al. (2011); whereas, “I would change life style if price of green products is less expensive”; and “I am willing to pay for green product if price are same” were adapted from Suki (2013). The CA coefficient was 0.80.

### Data Analysis

We adopt partial least squares structural equation modeling (PLS-SEM) as the statistical method to assess the research model in this study, using SmartPLS 3.3.3. PLS-SEM is primarily used to develop theories in exploratory research. It does this by focusing on explaining the variance in the dependent variables when examining the model. We employed PLS-SEM due to the inherent suitability of this approach for exploratory studies, which is the purpose of the current study (Ali et al., 2018).

PLS-SEM includes the evaluation of measurement model and structural model (Anderson and Gerbing, 1988). This combination analysis will enable the measurement errors of the observed variables to be analysed as a component of the model, and will combine the analysis of the variables of each factor with hypothesis testing.

In order to established the measurement model, the relationship between latent variables and their measurement items or indicators will be evaluated (Ali et al., 2018). In addition, the goodness of the measures used in the study also will be evaluated through indicator reliability and discriminant validity. The method includes checking the reliability of individual indicators, the internal consistency reliability of each construct, and the convergent and discriminant validity (Hair et al., 2017). For the next stage of the structural model, path coefficient analysis will be perform to explain the relationship between each latent variable. The model aims to test the predictive power of the research model (Peng and Lai, 2012). The model examines the endogenous variables (*R*^2^), the estimation of the path coefficient and a confidence interval of 95% (CI 0.95), and the estimation of the effect size (*f*^2^) (Hair et al., 2017).

## Results

### Measurement Model Assessment

This section indicates the criteria necessary to confirm the convergent validity, discriminant validity, and construct reliability of the measurement model. This study comprised of five reflective constructs, namely, ASS, BE, CPI, EA, and PP. In order to assess construct reliability, the threshold value of composite reliability (CR) and CA should be higher than 0.70. In this study, the values ranged from 0.861 to 0.949, thus confirming the construct reliability. Next, to assess the convergent validity, the threshold value of average variance extracted (AVE) and outer loadings should be more than 0.5 (Ali et al., 2018). The results of the AVE in this study ranged between 0.555 and 0.788, thus met the required thresholds. Furthermore, the outer loadings values are ranged between 0.675 and 0.925, were considered acceptable as they were above 0.50. Thus, it can be concluded that all constructs indicated an acceptable degree of convergent validity. The complete results are indicated in Table 2.

**TABLE 2:** Results of measurement model assessment.

Latent variable	Items	Loading	AVE	CR	CA	Mean	SD
ASS	ASS1	0.907	0.788	0.949	0.934	4.37	0.70
	ASS2	0.931					
	ASS3	0.852					
	ASS4	0.835					
	ASS5	0.909					
BE	BE1	0.900	0.734	0.932	0.909	3.85	0.79
	BE2	0.915					
	BE3	0.879					
	BE4	0.739					
	BE5	0.837					
CPI	PI1	0.889	0.749	0.937	0.916	4.23	0.67
	PI2	0.861					
	PI3	0.789					
	PI4	0.886					
	PI5	0.897					
EA	EA1	0.796	0.657	0.885	0.830	4.35	0.68
	EA2	0.805					
	EA3	0.790					
	EA4	0.851					
PP	P1	0.697	0.555	0.861	0.799	4.25	0.59
	P2	0.801					
	P3	0.794					
	P4	0.794					
	P5	0.665					

*ASS, after sales service; BE, brand equity; CPI, consumer purchase intention; EA, environmental awareness; PP, product pricing.*

We used the heterotrait-monotrait (HTMT) ratio to evaluate the discriminant validity of the research variables. For HTMT, discriminant validity is achieved when the correlation between each pair of the latent exogenous constructs is less than 0.85 (Henseler et al., 2015). Table 3 shows the value of HTMT for all constructs is lower than 0.85, therefore confirming discriminant validity.

**TABLE 3:** Discriminant validity using heterotrait-monotrait (HTMT) ratio.

	ASS	BE	CPI	EA	PP
ASS					
BE	0.242				
CPI	0.298	0.490			
EA	0.255	0.104	0.374		
PP	0.343	0.423	0.734	0.448	

*ASS, after sales service; BE, brand equity; CPI, consumer purchase intention; EA, environmental awareness; PP, product pricing.*

### Structural Model Assessment

Based on Table 2, the mean scores and standard deviations (SD) for our study variables were 4.20 for CPI (SD = 0.67); 4.35 for EA (SD = 0.68); 4.37 for ASS (SD = 0.70); 3.85 for BE (SD = 0.79); and 4.25 for PP (SD = 0.59). The collinearity between research variables was examined to ensure that the structural model did not include any lateral collinearity issue (Hair et al., 2017). Table 4 shows that all inner VIF values were below 5 (Hair et al., 2017), indicating that collinearity among the predictor constructs was not an issue in the structural model.

**TABLE 4:** Results of hypothesis testing.

Hypothesis	Relationship	Coefficient	*t*-value	95% CI	*f* ^2^	Supported	VIF
H1	ASS → CPI	0.051	0.746	[-0.086, 0.181]	0.004	No	1.149
H2	BE → CPI	0.256	3.577	[0.126, 0.406]	0.108	Yes	1.181
H3	EA → CPI	0.132	2.326	[0.023, 0.240]	0.028	Yes	1.200
H4	PP → CPI	0.482	7.278	[0.348, 0.605]	0.323	Yes	1.397

*ASS, after sales service; BE, brand equity; CPI, consumer purchase intention; EA, environmental awareness; PP, product pricing.*

**FIGURE 1:**
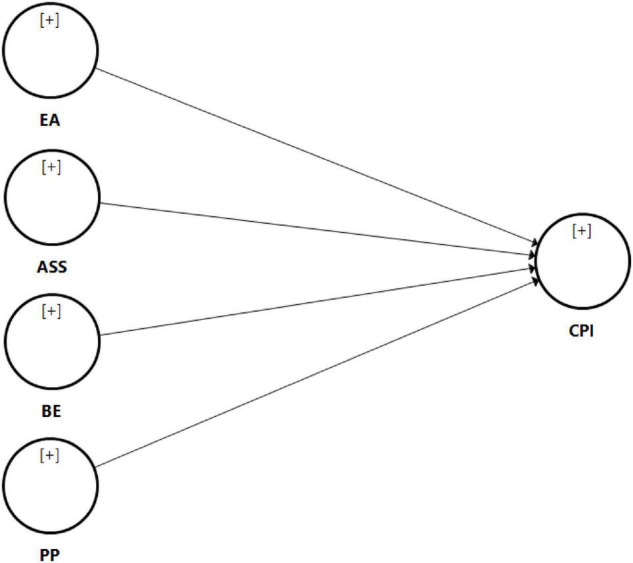
Results of assessment of structural model. ASS, after sales service; BE, brand equity; CPI, consumer purchase intention; EA, environmental awareness; PP, product pricing.

The results show a moderate, and substantial explanatory *R*^2^ value of 0.485 for CPI, suggesting that 48.5% of the variance for CPI can be described by ASS, BE, EA, and PP. The next step in the assessment of the structural model involves the evaluation of the path coefficients in relation to the model’s latent variables. Table 4 and Figure 1 indicate the results of the hypotheses testing, including the path coefficients and the effect size for each path. The results show that ASS (β = 0.051, *t* = 0.746, *p* > 0.05), BE (β = 0.256, *t* = 3.577, *p* < 0.05), EA (β = 0.132, *t* = 2.326, *p* < 0.05), and PP (β = 0.482, *t* = 7.278, *p* < 0.05) positively affects consumer purchasing intention, thus supporting hypotheses H2, H3, and H4. Meanwhile, ASS did not show a significant effect towards consumer purchasing intention, thus hypotheses H1 was rejected. PP has the strongest effect on CPI based on path coefficient and effect size, followed by BE and EA. Lastly, the results of cross validated redundancy indicate that the value of *Q*^2^ is greater than zero for the endogenous variables, which is 0.343 and acceptable (Ali et al., 2018). Therefore, the results allude to the predictive capability of the model based on the value of the endogenous constructs.

## Discussion

The main goal of this study is to determine whether ASS, BE, EA, and PP affected the consumer’s purchase intention on green home appliance brand in Penang, Malaysia. one hypothesis is rejected, and three hypotheses are accepted. PP is the strongest indicator of CPI, followed by BE and EA. Nevertheless, ASS appear to be not a significant predictor of CPI on green home appliance.

The result of H1 of this study was not supported, where ASS does not have significant effect on CPI. The research data show inconsistency with the research by Domazet et al. (2018); Huang and Ge (2019), and Singhal et al. (2019). Their result denotes that the responsiveness of ASS is one of the essential dimensions to be considered during the selection of home appliance by consumer. Nevertheless, research in other industries revealed similar outcome to the current study where ASS is insignificant to association of CPI. For example, Kesumahati and Jurnali (2020)’s study on pharmaceutical retail purchase exhibit that ASS quality does not has significant influence on the customer purchase intention. Similar finding is revealed by Akkucuk and Esmaeili (2016) in smartphone industry where BE and attributes are the primary consideration during purchase intention, and ASS is negligible. Both scenarios above can be explained by consumer is trusting on the branding of product or retail outlet. According to Aaker (1996), one of the dimensions in BE is consumer perceived quality. Perceived quality is not the actual quality of the product but the consumer’s subjective evaluation of the product (Jalilvand et al., 2011). In other words, if consumer trust on the branding of product, they will perceive that the product has good quality and subsequently neglect the actual ASS criteria. For example, if consumer purchase Apple product, they always assume the product quality and less defect. Hence, ASS is not an important issue.

The H2 of this research was that BE is positively affects CPI on green home appliance. The finding was supported therefore, our finding is aligned with Khan et al. (2015), Akkucuk and Esmaeili (2016), Vazifehdust et al. (2017), and Shafiee et al. (2019). Underpinning by Aaker’s (1996) Theory on BE, the researchers proved that strong brand awareness, brand association and perceived quality has strong impact on CPI. The positive impact of BE not only occur in green home appliance, but similar impact is also demonstrated across other industries like automobile, electronic gadget, fashion, food and beverage, etc. Hence, the manufactures of green home appliance shall focus on the marketing mix strategies to strengthen their BE. Studies evidenced that effective marketing mix strategies enable the company to develop a multi-dimensional consumer BE scale, especially in the area of brand awareness, perceived quality, and brand association (Valette-Florence et al., 2011; Soon-Ho and Lee, 2020).

The findings of this study also revealed the significant effect of EA on CPI on green home appliance in Penang, thus confirmed of those previous studies by Ahmed et al., 2019, Waris and Hameed (2020), and Teng et al. (2021). Where environmental consciousness has positively influence CPI on green product. Consumer who have higher environmental concern, usually will tend to purchase green product. Besides, the purchase intention of consumer will positively influence by green consciousness during their selection of product. The effort of cultivating the awareness and knowledge on environment need to be continued as there are proves showing ethical norms have a significant impact on the willingness to purchase of green appliance, however, this process consumes longer period and it need continuous effort, especially by government *via* education (Li et al., 2019).

Additionally, the findings confirmed the positive significant effect of PP on CPI on green home appliance in Penang, thus support the H4. Our findings corroborated previous studies by Akroush et al. (2019), Huang et al. (2019), and Li et al. (2019). The pricing factor can be positively influence the consumer intention or negatively impact on the consumer intention base on the price fairness concept (Haws and Bearden, 2006; Charuvatana, 2019). Consumers need to gain the benefits on additional price they spent on the green home appliance from various dimension. For example, buyer entitle for government income tax rebate if buying green home appliance. Another example would be consumer gain monetary advantage in utility consumption after investing in green home appliance. Without mentioned benefits, consumer will not intention to purchase green home appliance which is relatively higher price. Besides, our findings indicated that tax incentives, utility advantages, price reduction will stimulate their intention to purchase green home appliance. Moreover, household appliances are relatively expensive commodities and entail short and long-term financial consequences due to household budgets (Murali et al., 2016). Hence, the manufactures of green home appliance need to further examine their product cost structure, to provide a more affordable price to the income group of M40 and B40 in Penang, Malaysia. According to Tan et al. (2019), price is considered as the key factor affecting the buying decision of green products for price-sensitive customers.

Consumer purchase intention is a psychological process that is important to businesses and marketing professionals. A deep understanding of consumer intention help marketer to position the products and services effectively. If a consumer has a positive feeling on a specific product, it creates the purchase intention (Langga et al., 2020). Indubitably, our findings revealed that EA, BE, and pricing are imperative. In electrical home appliance, pricing and BE are three of the major consideration in various studies across the countries, which include Drozdenko et al. (2015) in United States, Shafiee et al. (2019) in Europe, and Wickramasinghe and Mathusinghe (2016) in Sri Lanka. The willingness of consumer to pay more for green product has decline even the concern on environmental has increase. Obviously, the main impediment is the personnel financial restriction especially during economic downturn (Drozdenko et al., 2015). Many scholars believe that the driving force to influence CPI in green home appliance is the product branding. The brand name itself is more than the generic product features and quality (Shafiee et al., 2019).

## Conclusion, Practical Implications, and Limitations

There were limited studies related to the factors that influenced consumers’ purchase intention on green home appliance in Malaysia (Ogiemwonyi et al., 2020; Harun et al., 2022), especially Penang. This study helps to examine the possible factors influence consumer intention of green home appliance brand in context of Penang consumer. The results will add value to the consumer purchasing behaviour and green marketing literature. Moreover, the findings of this research reveal that ASS have no significant influence on CPI on green home appliance brand. These findings have contradicted with some or previous research result in another region, and worthwhile to further investigate in Malaysia context.

Several practical implications also emerge. To attain the company business goal, the management need to understand the consumer intention, market trend, and demand for green home appliance. This has a robust relationship with the company marketing strategies which will influence consumers buying intention. Ultimately enhance company revenue. This research would help to provide home appliance manufacturers some insight and enables the manufactures to tailor the home alliance product which able to stimulate the purchase intention of consumer. The result of this research indicated that pricing is the primary factor which significantly impact the consumer intention. Therefore, manufacturers shall focus on reducing the green home PP, or the additional attributes of the green appliance shall be able to compensate the additional price occurred. Besides, the green home PP shall be designed in a more affordable position to cater 80% of Penang population which are income M40 and B40. BE is found to be another factor that influence CPI; therefore, the industries would need to devise better marketing strategies in Malaysia to enhance their brand image, brand awareness, and brand loyalty. As a result, it increases the sales and profit for the companies. Additionally, fossil fuel is the main source of electricity generation in Peninsular Malaysia, and household appliance are the main contributor of electricity consumption. By understanding the factors influencing consumer intention on green home appliance, government or related ministry can devise comprehensive strategy and policy to encourage the citizens, especially Penang citizens purchase intention of green appliance. For example, the Sustainable Energy Development Authority (SEDA) Malaysia had come up with Sustainability Achieved *Via* Energy Efficiency (SAVE) 3.0 programme as an initiative to increase public awareness to encourage them to buy energy efficient appliances that will save consumer’s electricity consumption, especially for domestic consumers. This programme is implemented from January 2022 to December 2022. The findings of this study can serve as insight for the next SAVE programme to encourage more households in Malaysia adopting green initiatives.

If pricing is the primary factor, government can provide convincing personal income tax rebate if the people purchase green home appliance. On the other hand, government can duplicate the green car policy in automobile industry where government waive the duties on green car.

There are several limitations that must be considered in this research. First, the research focuses only on four predictors which consist of EA, ASS, BE, and pricing on CPI. In fact, there should be more factors which influence CPI. Hence, future researchers can focus on other factors which are not mentioned in this study, for example, the demographical characteristics. Additionally, the concept of green home appliance in this study is macroscopic, but influencing factors may vary with the different types of product, for example, refrigerator, water heater, air conditioners, and other specific home appliance. Therefore, future research should focus on specific categories of energy-saving home appliance. Second, the data collection only can be done *via* online as Penang is under Movement Control Order 2.0 during the data collection. The time horizon for similar research can be perform base on longitudinal studies instead of cross-sectional. Hence, the causal and effect of target respondents can be observed, recorded, and analysed. For instance, collaboration with specified manufacturers to measure the impact of before and after price reduction on the sales volume of green home appliance. Finally, time constraint is another limitation. Hence, only 150 respondents able to be collected. If time permissible, higher amount of sample shall be collected to increase the accuracy and validity of the research result. Several other influencing factors could be investigated in future studies.

## Data Availability Statement

The raw data supporting the conclusions of this article will be made available by the authors, without undue reservation.

## Ethics Statement

Ethical approval was not provided for this study on human participants because the study was conducted according to the guidelines of the Declaration of Helsinki and following academic ethics. The patients/participants provided their written informed consent to participate in this study.

## Author Contributions

CT and KK: conceptualization, validation, and visualization. CT and WW: methodology. WW: software and formal analysis. CT: investigation, data curation, and writing—original draft preparation. CT, KK, and WW: resources and writing—review and editing. KK: supervision. All authors have read and agreed to the published version of the manuscript.

## Conflict of Interest

The authors declare that the research was conducted in the absence of any commercial or financial relationships that could be construed as a potential conflict of interest.

## Publisher’s Note

All claims expressed in this article are solely those of the authors and do not necessarily represent those of their affiliated organizations, or those of the publisher, the editors and the reviewers. Any product that may be evaluated in this article, or claim that may be made by its manufacturer, is not guaranteed or endorsed by the publisher.
